# Thresholds for unacceptable work state in radiographic axial spondyloarthritis of four presenteeism and two clinical outcome measurement instruments

**DOI:** 10.1093/rheumatology/keae033

**Published:** 2024-01-25

**Authors:** Dafne Capelusnik, Sofia Ramiro, Elena Nikiphorou, Walter P Maksymowych, Marina Nighat Magrey, Helena Marzo-Ortega, Annelies Boonen

**Affiliations:** Care and Public Health Research Institute (CAPHRI), Maastricht University, Maastricht, The Netherlands; Department of Rheumatology, Tel Aviv Sourasky Medical Center, Tel Aviv, Israel; Department of Rheumatology, Leiden University Medical Center, Leiden, The Netherlands; Department of Rheumatology, Zuyderland Medical Center, Heerlen, The Netherlands; Centre for Rheumatic Diseases, King’s College London, London, UK; Department of Rheumatology, King’s College Hospital, London, UK; Department of Medicine, University of Alberta, Edmonton, Alberta, Canada; Department of Rheumatology, Case Western Reserve University School of Medicine, Cleveland, OH, USA; NIHR Leeds Biomedical Research Centre, LTHT and LIRMM, University of Leeds, Leeds, UK; Care and Public Health Research Institute (CAPHRI), Maastricht University, Maastricht, The Netherlands; Department of Rheumatology, Maastricht University Medical Center, Maastricht, The Netherlands

**Keywords:** radiographic axial spondyloarthritis, work outcomes, presenteeism, work satisfaction

## Abstract

**Objectives:**

To (i) identify threshold values of presenteeism measurement instruments that reflect unacceptable work state in employed r-axSpA patients; (ii) determine whether those thresholds accurately predict future adverse work outcomes (AWO) (sick leave or short/long-term disability); (iii) evaluate the performance of traditional health-outcomes for r-axSpA; and (iv) explore whether thresholds are stable across contextual factors.

**Methods:**

Data from the multinational AS-PROSE study was used. Thresholds to determine whether patients consider themselves in an ‘unacceptable work state’ were calculated at baseline for four instruments assessing presenteeism and two health outcomes specific for r-axSpA. Different approaches derived from the receiver operating characteristic methodology were used. Validity of the optimal thresholds was tested across contextual factors and for predicting future AWO over 12 months.

**Results:**

Of 366 working patients, 15% reported an unacceptable work state; 6% experienced at least one AWO in 12 months. Optimal thresholds were: WPAI-presenteeism ≥40 (AUC 0.85), QQ-method <97 (0.76), WALS ≥0.75 (AUC 0.87), WLQ-25 ≥ 29 (AUC 0.85). BASDAI and BASFI performed similarly to the presenteeism instruments: ≥4.7 (AUC 0.82) and ≥3.5 (AUC 0.79), respectively. Thresholds for WALS and WLQ-25 were stable across contextual factors, while for all other instruments they overestimated unacceptable work state in lower educated persons. Proposed thresholds could also predict future AWO, although with lower performance, especially for QQ-method, BASDAI and BASFI.

**Conclusions:**

Thresholds of measurement instruments for presenteeism and health status to identify unacceptable work state have been established. These thresholds can help in daily clinical practice to provide work-related support to r-axSpA patients at risk for AWO.

Rheumatology key messages

- Presenteeism thresholds identifying persons with current unacceptable work state are: WPAI-presenteeism ≥40; QQ-method <7; WALS ≥0.75; and WLQ-25 ≥29.
- BASDAI ≥4.7 and BASFI ≥3.5 can be used to accurately identify patients with an acceptable work state.
- Except for WLQ-25 and WALS, proposed thresholds overestimated unacceptable work state for lower-educated patients.

## Introduction

Axial spondyloarthritis (axSpA) has an early disease onset and typically affects young people of working age [1]. Many studies during the last two decades have demonstrated the impact of axSpA on work participation and its consequences on costs of lost productivity and life satisfaction [2, 3]. While new treatment options and better control of disease activity have improved work participation, an employment gap compared with the general population remains, suggesting that further support is still needed [4–6].

Work participation (WP) refers to active engagement in paid work, which distinguishes absenteeism and presenteeism. Absenteeism refers to the time missed from work due to health reasons (i.e. sick leave or work disability) and presenteeism to the reduction in work ability or productivity while at work, due to health reasons [7, 8].

Currently, efforts to improve WP aim to prevent withdrawal from the labour force, as return to work in case of work disability is unlikely. Moreover, there is broad consensus that staying at work has longer-term benefits on physical and mental health, but also on economic self-sufficiency and societal costs [9]. It has been already shown that the occurrence of previous sick leave, in the general population and in axSpA is a strong predictor of future prolonged sick leave [10] with the ultimate risk of withdrawal from paid work [11]. Independent of past sick leave and health impairments, presenteeism is also a strong predictor of future long-term sick leave [12–16]. On that line, presenteeism can be an interesting measure to select persons with risk for future prolonged sick leave and potentially work disability. In research on WP, the role of context to influence outcome or modify effect of interventions is increasingly recognized [17]. Specifically in axSpA, age, nature of work (physical work) and a lower educational level were found to be associated with sick leave and higher presenteeism [15, 18].

From a large number of available instruments to assess presenteeism, the Outcome Measures in Rheumatology (OMERACT) [19] endorsed two instruments that passed the filter of validity: the presenteeism question of the Work Productivity and Activity Impairment questionnaire (WPAI) and the RA-specific Work Productivity Survey (WPS-RA). Additionally, the Quality and Quantity method (QQ method), Workplace Activity Limitations Scale (WALS) and Work Limitations Questionnaire-25 (WLQ-25) were also selected as candidate instruments and are currently being assessed for their measurement properties (truth, discrimination and feasibility) to fulfill the OMERACT filter.

Despite some lower precision, categorical variables are more practical to be used, easier to interpret, thus more applicable to clinical practice. Threshold of meaning can refer to the relevant absolute change, like the minimal important difference (MID) but also could be relevant and useful to determine thresholds of presenteeism reflecting an unacceptable work state. Comparative data on the threshold for unacceptable work state in axSpA are missing for the OMERACT candidates. Also, despite the recognition of the importance of contextual factors, the role of contextual factors in measurement properties of those instruments was not sufficiently explored. Notwithstanding, this is relevant to understand whether different thresholds should be applied in different subgroups of patents.

Our aim was to identify the thresholds of four recommended instruments for presenteeism that reflect most accurately a patient’s health state that would be considered as an unacceptable work state. Additionally, we aimed to explore whether these thresholds predict future adverse work outcome (AWO) and whether they are stable across contextual factors. Cut-off values for traditional outcome instruments, namely BASDAI and BASFI were also studied, to explore whether they provide equally accurate information regarding acceptable work state or future AWO, as this might facilitate identification of persons at risk in daily practice when no presenteeism instruments are available.

## Methods

### Study design and patient recruitment

Data from the multinational, prospective observational study on Patient-Reported Outcomes Survey of Employment in Ankylosing Spondylitis (AS-PROSE) were used. Briefly, the AS-PROSE study includes data on WP in patients with radiographic axSpA (r-axSpA) according to their treating rheumatologists, from four countries: Canada, the Netherlands, United Kingdom and United States. Data were collected from October 2008 to December 2013. Written informed consent was obtained from all patients before enrolment and ethics committees from the individual participating centres were involved in approving the study and the ethics committee from the University of Alberta approved this project (ID Pro00123727). For the current study, data of patients between 18 and 65 years old (working age), at work at the moment of the first visit was used.

### Data collection

Participants were asked to complete an online survey every three months during a one-year period (baseline, 3, 6, 9 and 12 months). The data collected included socio-demographics at baseline [e.g. age, gender, ethnicity, education (superior: university or tertiary education / no-superior education: primary or secondary education), marital status] and at each visit information on work (i.e. being employed or not, presenteeism, sick leave), axSpA characteristics [i.e. symptom and disease duration, current or past presence (ever) of peripheral arthritis], health outcomes [e.g. BASFI, BASDAI, several instruments to measure health-related quality of life, patient-acceptable symptom state (PASS) [20]], work-related context (e.g. nature of the work or job type, dichotomized into blue or white collar, and full or part-time work) and lifestyle factors [i.e. body mass index (BMI), past or current smoking].

#### Instruments for which thresholds were determined

Four self-reported instruments addressing presenteeism or including presenteeism as a sub-scale were included in AS-PROSE. Two assess the global impact of health on work (WPAI presenteeism subscale and QQ method) and two are multi-item, multi-dimensional instruments (WALS and WLQ-25) which address the impact of health on various aspects of work [19, 21–24]. The WPAI presenteeism scores limitations in productivity while at work (0–100%; 100=worst productivity); the QQ method combines global assessment of quality and quantity of work (0–10; 10=best quality and quantity), the WALS contains 12 questions on the degree of (dis)ability related to work (0–3; 3=worst ability) and the WLQ-25 has 25 items across four subscales addressing the percentage of time at work with various limitations [each scale ranges from a 0–100 scale (maximally limited)]. The four subscales can also be converted into the WLQ-25 by calculating the average of the four of them and providing the estimated productivity loss (0–100; 100=maximal productivity loss) [25]. A more detailed description of the instruments is shown in Supplementary Text S1, available at *Rheumatology* online.

In addition, the threshold values that indicate unacceptable work state were determined for the Bath Ankylosing Spondylitis Disease Activity Index (BASDAI) [26] and the Bath Ankylosing Spondylitis Functional Index (BASFI) [27].

#### Anchor questions

Two predefined external criteria were chosen due to being considered relevant when implementing thresholds in clinical practice. First, the AS-PROSE case report form (CRF) included a question on whether patients considered themselves in an acceptable work state [Patient Acceptable Work State (PAWS)] through the question: ‘Considering all the different ways your disease is affecting you, if you would stay in this state for the next few months, do you consider that: your ability to perform your current job is satisfactory?’. To align the direction of the anchor question with the presenteeism instruments, the scale was inverted so that measurement of an unacceptable work state (yes/no) could be possible. Second, as a long-term outcome, future AWO over the 12 months of follow-up was used, defined as any sick leave, short- or long-term disability.

### Statistical analysis

#### Threshold analyses

Persons with paid work at baseline and not on sick leave were considered. Receiver operating characteristic (ROC) analysis was used to define the thresholds of meaning [28]. This statistical approach permits the determination of the threshold with a preferred balance between sensitivity (SE) and specificity (SP) for each measurement instrument. We applied four approaches to find the optimal cut off: (i) 75% percentile method, which is the 75th percentile of the distribution of the presenteeism scores; in order to favour more sensible thresholds, we calculated this in patients who considered themselves in an acceptable work state, so that a value above the cutoff reflects an unacceptable work state. This approach has been validated as a comparable alternative to the ROC curve analysis, and is much easier to derive [29, 30]; (ii) Liu method, that maximizes the product of the SE and SP [31]; (iii) the Youden index, assumes that sensitivity and specificity are equally important and subtracts the value 1.0 from the sum of sensitivity and specificity, so that the maximum value of the index becomes 1 when there is perfect agreement [32]; and (iv) the nearest to 0.1, which is the point where the shoulder of the ROC curve is closest to the left upper corner of the graph (the point with optimal SE and SP) [33]. Because the last three methods balance SE and SP, we added an alternative ROC-based approach in which we gave more weight to SE and higher positive likelihood ratio (LR+), taking into account that there are limited consequences of an ‘overdiagnosis’ (i.e. low specificity) for patients and the healthcare system and that we consciously want to be sensitive and identify patients at risk. When two thresholds seemed to perform well, the decision was based on the performance, i.e. proportion correctly classified combined with absolute numbers of false positives compared with false negatives of the candidate thresholds as described below. We also adapted, when necessary, theoretical numbers from the output of the different methods to the closer real number of the scale with the same SE and SP.

#### Validation analysis

A validation analysis was performed to assess the performance of the selected thresholds across different sub-populations. First, a temporal validation was performed among persons with paid work at 12 months. Next, we tested the performance of the thresholds in subgroups of patients defined by contextual factors being by age, gender, job type (white or blue-collar), ever peripheral involvement (pure axial or axial and peripheral), superior education and BMI. For continuous variables, the median value was used to dichotomize the population. We compared the proportion of patients correctly classified as being in an ‘unacceptable work state’ as well as the proportion of patients who were under-estimated or over-estimated. The thresholds performance was considered stable if there was no relevant difference when judging accuracy (<10%) between the two subpopulations.

The thresholds for the presenteeism instruments were also validated against AWO during the 12 months of follow-up. Finally, thresholds for the four subscales of the WLQ-25 were determined, as these are regularly also reported in practice.

All the analyses were performed using Stata SE V.14.

## Results

### Baseline characteristics

Of 555 patients participating in AS-PROSE, 366 (66%) were included in the current analyses as they were 65 years or younger, had paid work at baseline and were not on sick leave. Of these, 72% were men, with a mean (SD) age of 43 (10) years and a mean symptom duration of 18 (11) years. Mean baseline BASDAI and BASFI were 4.0 (2.1) and 3.5 (2.2), respectively, and 45% of the patients were on treatment with biologic disease-modified antirheumatic drugs (bDMARDs). A total of 79% of the patients had a full-time job and 26% were blue-collar workers. Baseline characteristics for the total population as well as stratified by satisfaction with work state are shown in Table 1. Supplementary Tables S1 and S2 (available at *Rheumatology* online) provide a detailed description by origin country and by presence of AWO during the 12-month follow-up.

**Table 1.** Baseline characteristics of the total population and by un/acceptable work state

	Total N = 366	Acceptable work state N = 302	Unacceptable work state N = 55
Age, years	43 (10)	43 (10)	44 (11)
Gender, male	263 (72)	217 (72)	42 (76)
Caucasian race, yes	337 (92)	281 (93)	52 (95)
Symptom duration, years^a^	18.2 (11.0)	18.4 (10.9)	17.9 (11.9)
Disease duration, years^a^	11.6 (10.0)	12.0 (10.1)	10.1 (9.8)
Pure axSpA	100 (27)	90 (30)	8 (15)
BMI, kg/m^2^^b^	28.1 (6.9)	27.9 (6.9)	29.9 (6.7)
Smoking status			
Never	185 (51)	157 (52)	24 (44)
Past	128 (35)	107 (35)	18 (33)
Current	53 (14)	38 (13)	13 (24)
Marital status			
Single	61 (17)	47 (16)	11 (20)
Married/partner	274 (75)	230 (76)	39 (72)
Divorced/widowed	30 (8)	25 (8)	4 (18)
Education			
Primary	15 (4)	11 (4)	4 (7)
Secondary	60 (17)	46 (15)	14 (25)
Technical school	114 (31)	92 (30)	20 (36)
University	117 (48)	153 (51)	17 (31)
Blue-collar worker, yes	95 (26)	73 (25)	22 (42)
bDMARDs at baseline	250 (45)	144 (48)	27 (49)
BASDAI (0-10)	4.0 (2.1)	3.6 (1.9)	6.1 (1.8)
BASFI (0-10)	3.5 (2.2)	3.0 (1.9)	5.7 (2.4)
WPAI presenteeism (0–100%)	25.0 (24.6)	19.1 (18.8)	55.6 (28.7)
QQ method (1–100)	78.3 (27.2)	83.8 (22.8)	52.2 (32.0)
WALS (0–3)	0.62 (0.46)	0.50 (0.37)	1.21 (0.44)
WLQ-25 (0–100)	22.7 (17.7)	18.8 (14.3)	44.6 (18.1)
Adverse work outcome^c^	22 (6)	9 (3)	12 (22)

Patients ≤65 years old, employed, not on sick leave at baseline (second column) who had completed the PAWS question (third and fourth columns).

Results reflect mean (SD) or *n* (%).

a <5% missing data.

b <10% missing data.

c Adverse work outcome including sick leave, short term disability and long-term disability.

axSpA: axial spondyloarthritis; BASDAI: Bath Ankylosing Spondylitis Disease Activity Index; BASFI: Bath Ankylosing Spondylitis Functional Index; bDMARDs: biologic disease modified antirheumatic drugs; BMI: body mass index; QQ method: quantity and quality method; WALS: Workplace Activity Limitations Scale; WLQ-25: Work Limitations Questionnaire; WPAI: Work Productivity and Activity Impairment.

The mean baseline values of the four presenteeism instruments were: WPAI-presenteeism, 25.0 (24.6); QQ method, 78.3 (27.2); WALS, 0.62 (0.46); and WLQ-25, 22.7 (17.7). Each instrument distribution can be found in the Supplementary Fig. S1, available at *Rheumatology* online. At baseline, 15% (*n* = 55) of the patients were unsatisfied with their work state (PAWS anchor question), and 6% (*n* = 22) already reported an AWO at baseline (Supplementary Table S3, available at *Rheumatology* online).

After 12 months of follow-up, 65% (*n* = 226) of the working patients remained in the same work status, and 27% (*n* = 84) from the patients not working at baseline, remained without work. During that period, 5% patients (*n* = 19) of those with paid work changed their status to ‘not working’, and 3% (*n* = 11) transitioned from ‘not working’ to ‘paid work’.

### Thresholds for ‘unacceptable work state’

**Figure 1.**
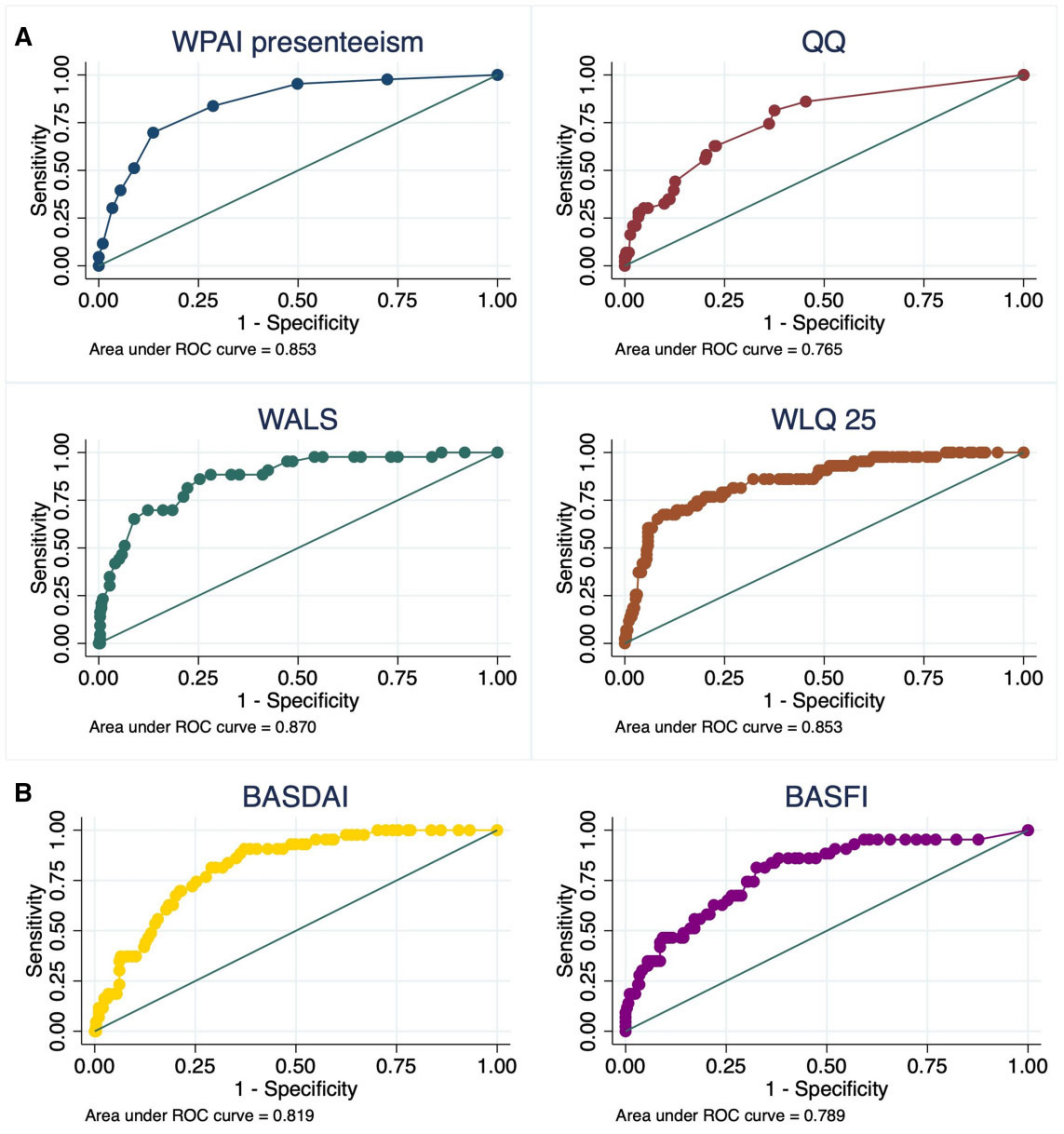
(**A**) ROC curves for excessive presenteeism from different measurement instruments according to unacceptable work state. QQ method: quantity and quality method; ROC: receiver operating characteristic; WALS: Workplace Activity Limitations Scale; WLQ-25: Work Limitations Questionnaire; WPAI: Work Productivity and Activity Impairment. (**B**) ROC curves for specific health state measurement instruments according to unacceptable work state. BASDAI: Bath Ankylosing Spondylitis Disease Activity Index; BASFI: Bath Ankylosing Spondylitis Functional Index; ROC: receiver operating characteristic

In general, the Liu and the nearest to 0.1 ROC methods were considered the most appropriate criterions to discriminate between patients with unacceptable opposed to acceptable work state: when applying the exercise of calculating the proportion of correctly classified cases, with absolute numbers of false positives compared with false negatives, the thresholds from the Liu method and the nearest to 0.1 method were the ones with better performance. The finally selected optimal thresholds for each measurement instrument with proportion of persons (in)correctly classified, SE/SP and AUC are presented in Table 2. The AUC by ROC for all the instruments were >0.80, with the exception of the QQ method (AUC 0.765). BASDAI and BASFI performed similarly to the presenteeism instruments (Fig. 1A and B). Supplementary Table S4, available at *Rheumatology* online presents, additionally to the selected optimal threshold, the results for the different ROC methods (thresholds, AUC, SE/SP) for each measurement instrument. After the dichotomization of the measurement instruments by our proposed threshold value, the AUC remained acceptable, although again with lower values for the QQ method and BASFI.

**Table 2.** Performance assessment of each presenteeism instrument when classifying unacceptable work state

		PAWS acceptable work state	PAWS not acceptable work state	Correctly classified	Over-estimated	Under-estimated	Selected threshold (SE/SP) [AUC]
WPAI presenteeism ≥30^a^	Acceptable (<30)	209 (97)	7 (3)	245 (73)	84 (25)	7 (2)	40 (70/86) [0.853 (0.794–0.911)]
	Unacceptable (≥30)	84 (70)	36 (30)				
WPAI presenteeism ≥40^a^	Acceptable (<40)	253 (95)	13 (5)	283 (83)	40 (12)	13 (4)	
	Unacceptable (≥40)	40 (57)	30 (43)				
QQ method <97	Acceptable (≥97)	183 (96)	8 (4)	218 (65)	110 (33)	8 (2)	97 (81/62) [0.765 (0.690–0.840)]
	Unacceptable (<97)	110 (76)	35 (24)				
WALS ≥0.75	Acceptable (<0.75)	218 (97)	6 (3)	255 (76)	75 (22)	6 (2)	0.75 (86/75) [0.870 (0.813-0.926)]
	Unacceptable (≥0.75)	75 (67)	37 (33)				
WLQ 25 ≥ 29	Acceptable (<29)	233 (96)	10 (4)	266 (79)	59 (18)	10 (3)	29 (77/80) [0.853 (0.790–0.915)]
	Unacceptable (≥29)	59 (64)	33 (36)				
BASDAI ≥4^b^	Acceptable (<4)	184 (98)	4 (2)	223 (66)	109 (32)	4 (1)	4.7 (81/71) [0.819 (0.762–0.876)]
	Unacceptable (≥4)	109 (74)	39 (26)				
BASDAI ≥4.7^b^	Acceptable (<4.7)	212 (96)	10 (4)	245 (73)	81 (24)	10 (4)	
	Unacceptable (≥4.7)	81 (71)	33 (29)				
BASFI ≥3.5	Acceptable (<3.5)	197 (96)	8 (4)	232 (69)	95 (28)	8 (2)	3.5 (81/67) [0.789 (0.715–0.862)]
	Unacceptable (≥3.5)	95 (73)	35 (27)				

*n* (%), number of patients, unless otherwise stated.

a Performance comparison between the WPAI presenteeism threshold 30 *vs* 40.

b Performance comparison between the BASDAI threshold 4 *vs* 4.7.

BASDAI: Bath Ankylosing Spondylitis Disease Activity Index; BASFI: Bath Ankylosing Spondylitis Functional Index; QQ method: quantity and quality method; WALS: Workplace Activity Limitations Scale; WLQ-25: Work Limitations Questionnaire; WPAI: Work Productivity and Activity Impairment.

When assessing individual thresholds for each WLQ-25 subscale, the thresholds for the mental-interpersonal demands and output demands were slightly lower (17 and 20). Moreover, regarding the physical demands subscale, the optimal thresholds varied considerably across the different methods (data not shown). However, when comparing the performance of each subscale obtained thresholds and the overall WLQ-25 threshold applied to each individual subscale, in all the cases the thresholds for the average of the four scale WLQ-25 worked better.

### Validation of thresholds

When repeating the analyses with data from the timepoint 12 months (Table 3), no important variation (<10%) was found between the percentage of correctly classified patients compared with baseline (main analysis).

**Table 3.** Validation analysis across different populations. Percentages of patients correctly classified according to the selected thresholds

	Time point		Age (years)		Gender		Job type		Type of disease		Education		BMI		Symptom duration	
	Baseline (*n* = 366)	12 months (*n* = 221)	≤43 (*n* = 164)	>43 (*n* = 172)	Male (*n* = 172)	Female (*n* = 93)	Blue-collar (*n* = 85)	White- collar (*n* = 244)	Pure axial (*n* = 94)	Mixed (*n* = 242)	No superior education (*n* = 66)	Superior education (*n* = 270)	BMI ≤28 (*n* = 189)	BMI >28 (*n* = 147)	<18 years symptom duration (*n* = 169)	≥18 years symptom duration (*n* = 175)
WPAI presenteeism ≥40	283 (83)	185 (84)	143 (87)	140 (81)	211 (87)	72 (77)	65 (76)	212 (87)	79 (84)	204 (84)	49 (74)	234 (87)	160 (85)	123 (84)	137 (84)	146 (84)
QQ method <97	218 (65)	141 (64)	108 (66)	110 (64)	166 (68)	52 (56)	46 (54)	165 (68)	63 (67)	155 (64)	35 (53)	183 (68)	120 (63)	98 (67)	101 (62)	117 (68)
WALS ≥0.75	255 (76)	177 (80)	129 (79)	126 (73)	189 (78)	66 (71)	66 (78)	185 (76)	75 (80)	180 (74)	51 (77)	204 (76)	149 (79)	106 (72)	119 (73)	136 (79)
WLQ-25 ≥ 29	266 (79)	178 (81)	131 (80)	135 (78)	188 (77)	78 (85)	66 (78)	196 (81)	76 (81)	190 (79)	48 (73)	218 (81)	155 (82)	111 (76)	122 (75)	144 (83)
BASDAI ≥4.7	245 (73)	175 (81)	115 (70)	130 (76)	185 (76)	60 (65)	59 (69)	181 (74)	75 (80)	170 (70)	45 (68)	200 (74)	134 (71)	111 (76)	117 (72)	128 (74)
BASFI ≥3.5	232 (69)	163 (75)	124 (76)	108 (63)	176 (72)	56 (61)	58 (68)	169 (70)	69 (73)	163 (68)	39 (60)	193 (71)	137 (72)	95 (65)	119 (73)	113 (66)

*n* (%), number of patients, unless otherwise stated.

Shaded cells correspond to stratifications where the percentage of correctly classified patients between the subgroups vary in ≥10%.

BASDAI: Bath Ankylosing Spondylitis Disease Activity Index; BASFI: Bath Ankylosing Spondylitis Functional Index; QQ method: quantity and quality method; WALS: Workplace Activity Limitations Scale; WLQ-25: Work Limitations Questionnaire; WPAI: Work Productivity and Activity Impairment.

Across contextual factor subgroups, the multidimensional presenteeism instruments (WALS and WLQ-25), were consistently stable across all the external factors examined. However, for all other instruments evaluated, the proportion of persons correctly classified differed by at least 10% for subgroups defined by sex and education where the proposed threshold overestimated unacceptable work state among female patients, lower-educated persons and blue-collar workers. When further exploring the role of sex, further analyses revealed the proportion of males with full-time work to be higher than in females (88% *vs* 56%). Subsequently, when applying the thresholds only in those patients with a full-time job, there were no longer differences in the thresholds between males and females. Additionally, job type influenced the threshold for the global presenteeism instruments (WPAI and QQ) with thresholds again mainly overestimating (but also slightly underestimating) unacceptable work state (Supplementary Table S5, available at *Rheumatology* online). Full performance data from each subgroup can be found in Supplementary Tables S6–S13, available at *Rheumatology* online.

When applying the selected thresholds at baseline to predict an AWO during 12 months, thresholds for WPAI presenteeism, WALS and WLQ-25 performed slightly lower (68–76% correctly classified) compared with the 76–83% from the main analysis, identifying those with a current unacceptable work state. BASDAI and BASFI thresholds had even worse performance (66–67% and 73–69%, respectively, for identifying current unacceptable work state) and the threshold for the QQ method also performed clearly worse (58%) (Table 4).

**Table 4.** Performance of the threshold for each presenteeism instrument in predicting adverse work outcome over 12 months

Presenteeism	No adverse work outcome	Adverse work outcome	Correctly classified	Over-estimated	Under-estimated	Selected threshold AUC [SE/SP]
WPAI <40	207 (94)	13 (6)	215 (76)	55 (19)	13 (5)	0.625 [38/79]
WPAI ≥40	55 (87)	8 (13)				
QQ method ≥97	148 (94)	9 (6)	160 (57)	114 (40)	9 (3)	0.598 [57/56]
QQ method <97	114 (90)	12 (10)				
WALS <0.75	181 (95)	9 (5)	193 (68)	81 (29)	9 (3)	0.712 [57/69]
WALS ≥0.75	81 (87)	12 (13)				
WLQ-25 < 29	192 (94)	12 (6)	201 (71)	69 (24)	12 (4)	0.617 [57/74]
WLQ-25 ≥ 29	69 (88)	9 (12)				
BASDAI <4.7	178 (95)	9 (5)	290 (67)	84 (30)	9 (3)	0.663 [57/68]
BASDAI ≥4.7	84 (88)	12 (12)				
BASFI <3.5	170 (97)	6 (3)	185 (66)	91 (32)	6 (2)	0.715 [71/65]
BASFI ≥3.5	91 (86)	15 (14)				

*n* (%), number of patients, unless otherwise stated.

BASDAI: Bath Ankylosing Spondylitis Disease Activity Index; BASFI: Bath Ankylosing Spondylitis Functional Index; QQ method: quantity and quality method; WALS: Workplace Activity Limitations Scale; WLQ-25: Work Limitations Questionnaire; WPAI: Work Productivity and Activity Impairment.

## Discussion

In this study, we established thresholds for four presenteeism measurement instruments and two disease-specific health outcomes to identify r-axSpA working patients that experience an unacceptable work state. Overall, the proposed thresholds performed well in terms of accurately classifying patients and were acceptable in predicting AWO over 12 months.

The proposed thresholds for the presenteeism instruments are ≥40 for WPAI-presenteeism, <97 for the QQ-method, ≥0.75 WALS and ≥29 for the WLQ-index. Additionally, thresholds values for unacceptable work state were ≥4.7 for BASDAI and ≥3.5 for BASFI. The QQ method demonstrated the poorest performance out of the four presenteeism measurement instruments with only 65% of patients correctly classified. The other three had similar performance, although slightly better accuracy (83%) for the WPAI presenteeism *vs* 79% for the WLQ-25 and 76% for the WALS. The lower performance of the QQ method is in line with its lower evidence of content validity in comparison with the other instruments [19]; the fact that the recall period in the questions refers to ‘last workday’, could contribute to its less accurate results. Additionally, the distribution of the instrument (highly right-skewed) could also influence this lower accuracy of the thresholds. The favorable performance of the thresholds for the WPAI presenteeism question is of particular interest, as the WPAI is frequently used in clinical studies and increasingly in clinical practice [34].

For the WLQ-25, we focused on the frequently used average of the four subscales but also calculated the thresholds for each subscale in an additional analysis. For the subscale physical demand, the threshold was unstable across the four statistical methods (75 for the 75th percentile approach, 31 for the Liu method and nearest to 0.1 method and 23 for the Youden index). Likely, the reversed direction when answering the questions in this subscale compared with the others was not adequately picked up by patients. This is a well-known limitation of the WLQ-25, hampering its ability to provide a reliable threshold for ‘acceptable work state’ for this subscale. A modification of the WLQ-25 has therefore been proposed, but to the best of our knowledge it has not yet been widely implemented [19]. Data for our study was collected before this modification and we could not include it.

An interesting point of the present study is that thresholds to identify patients in an unacceptable work state were not only established for presenteeism instruments, but also for two traditional disease outcomes, namely BASDAI and BASFI, which proved to have a similar performance when identifying patients in an unacceptable work state. This finding is of great significance due to the more widespread use of the BASDAI and BASFI, particularly in daily clinical practice, in contrast with the presenteeism measurement instruments.

The availability of thresholds will enable the identification of persons with problematic work state in clinical practice as well as in research. However, it should be acknowledged that the use of a categorical as opposed to a continuous scale will inevitably result in lower precision. As we favoured sensitivity, our proposed threshold could result in some overestimation. This was a conscious choice as further understanding of the specific work-related issues and the required support are neither extremely time consuming nor expensive while potentially effective in keeping patients at work. Tailored interventions to these selected candidates can help them (re)achieve an acceptable work state.

Across contextual factors, the accuracy of thresholds for both multi-dimensional presenteeism questionnaires (WALS and WLQ-25) remained unchanged. However, thresholds of all other measurement instruments were sensitive to educational level and the QQ and WPAI also for job type. Lower educated persons and blue-collar workers risked being overestimated. It should be noted that these findings might be confounded by internal (work related) and external (not work related) factors that were not taken into account. For example, the differences in accuracy between sex disappeared after the exclusion of patients with part-time work (more frequent among females). The greater stability demonstrated by WALS and WLQ-25, two multi-dimensional instruments, is not surprising, as it is likely the questions on the specific work challenges account for context.

Unfortunately, the thresholds had low accuracy in predicting AWO in the following year, and this was especially the case for the QQ method, followed by the two traditional outcomes, BASDAI and BASFI. Clearly, future AWO is dependent on many other factors. Notwithstanding, having some threshold to identify unacceptable work state can be combined with other predictors to evaluate the level of risk for AWO and intensity of support [10].

While the AS PROSE study included data with three variables collected independently (sick leave, short-term and long-term disability) no clear definition for each one of them was provided in the questionnaire, and therefore this could lead to a different interpretation of their meaning across the different participating countries. To overcome this issue, in the present study, the term AWO was used, representing the three different outcomes together.

In the literature, thresholds for minimal clinically important differences (MCIDs) have been defined for several presenteeism measurement instruments. For example, the MCID for WPAI presenteeism in patients with psoriatic arthritis was estimated to be 20% [17, 35]. However, literature on an acceptable state of presenteeism that is ‘satisfactory’ and comparison across instruments is rare and has not been specifically conducted in axSpA. In view of the limited number of studies, and despite a temporal validation of the thresholds in this study, i.e. they were similar at baseline and 12 months, these thresholds should preferably be further validated in other studies to confirm their generalizability.

In summary, stable thresholds for presenteeism instruments representing unacceptable work state, irrespective of contextual factors, have been established for r-axSpA: ≥40 for WPAI presenteeism, <97 for the QQ method, ≥0.75 WALS and ≥29 for the WLQ index. Interestingly, similar performance has been demonstrated by more traditional instruments, namely BASDAI and BASFI (≥4.7 for BASDAI and ≥3.5 for BASFI), which can also be used in daily clinical practice to signal patients benefiting from more tailored interventions to maximize their chances of staying at work.

## Supplementary material

Supplementary material is available at *Rheumatology* online.

## Supplementary Material

keae033_Supplementary_Data

## Data Availability

The data underlying this article will be shared on reasonable request to the corresponding author.
