# Supplementary material for: Thresholds for unacceptable work state in radiographic axial spondyloarthritis of four presenteeism and two clinical outcome measurement instruments
Source: Rheumatology (Oxford). 2024 Jan 25;64(1):358–66. doi: 10.1093/rheumatology/keae033 (PMC11701323; doi:10.1093/rheumatology/keae033)
Supplement: keae033_Supplementary_Data [file keae033_supplementary_data.docx]

**Thresholds for unacceptable work state in axial spondyloarthritis of four presenteeism and two clinical measurement instruments**

**Supplementary material:**

**Supplementary Text S1**

**Explanation and calculation of the presenteeism measurement instruments**

Detailed on the calculation of scale scores for each scale is provided below.

***Work Productivity and Activity Impairment questionnaire (WPAI)***(1)

**Q1:** Current employment status

**Q2:** Number of hours missed due to health problem

**Q3:** Number of hours missed due to other reasons

**Q4:** Number of hours actually worked

**Q5:** Degree to which health affected productivity while working (Presenteeism)

**Q6:** Degree to which health affected regular (nonwork) activities

Percentage impairment while working because of problem (percentage of presenteeism):

- - How much AS affected work **(Q5** / 10) *100

***Quantity and Quality Method (QQ-method)***(2)

- - Final QQ -method = Quantity Loss*Quality Loss

***Workplace Activity Limitations Scale (WALS)***(3)

- Average across the 12 items, resulting in a range from 0 – 3

***Work Limitations Questionnaire (WLQ-25)***(4,5)

- - Reverse score items on Physical Demands (PD) subscale so that higher scores reflect more limitations
  - Overall Scale Score:
    - Multiply the mean of all 25 items by 25, resulting in a range of 0 (limited none of the time) – 100 (limited all of the time)
  - Four Subscales
    - Time management (TM) – Multiply the mean of 5 TM items by 25
    - Physical demands (PD) – Multiply the mean of 6 PD items by 25
    - Mental-interpersonal demands (MI) – Multiply the mean of 9 MI items by 25

Output demands (OD) – Multiply the mean of 5 OD items by 25

**References**

1. Reilly MC, Zborzef AS, Dukes EM. The validity and reproducibility of a work productivity and activity impairment instrument. Pharmacoeconomics 1993;4:353-65
2. Brouwer WB, Koopmanschap MA, Rutten FF. Productivity losses without absence: measurement validation and empirical evidence. Health Policy 1999;48:13-27
3. Gignac MA. Arthritis and employment: and examination of behavioral coping efforts to manage workplace activity limitations. Arthritis Rheum 2005;53:328-36
4. Lerner D, Amick BC III, Rogers WH, Malspeis S, Bungay K, Cynn D. The Work Limitations Questionnaire. Med Care 2001;39:72-85

| **Supplementary Table S1. Baseline characteristics in the total population and by country** | | | | | |
| --- | --- | --- | --- | --- | --- |
|  | Total  N=366 | CA  N=175 | UK  N=77-92 | NL  N=74 | US  N=25 |
| Age, years | 43 (10) | 43 (10) | 42 (10) | 46 (10) | 43 (12) |
| Gender, male | 263 (72) | 128 (73) | 63 (68) | 56 (76) | 16 (64) |
| Caucasian race, yes | 337 (92) | 159 (91) | 86 (93) | 70 (95) | 22 (88) |
| Symptom duration, years^#^ | 18.2 (11.0) | 18.2 (10.4) | 15.6 (11.5) | 22.2 (11.0) | 15.7 (9.4) |
| Disease duration, years^#^ | 11.6 (10.0) | 11.3 (9.6) | 9.9 (10.3) | 15.1 (10.1) | 9.8 (9.6) |
| Pure axSpA | 100 (27) | 43 (25) | 22 (24) | 28 (38) | 7 (28) |
| BMI, kg/m^2 ##^ | 28.1 (6.9) | 28.8 (8.1) | 27.4 (5.7) | 26.0 (3.8) | 31.3 (6.5) |
| *Smoking status* |  |  |  |  |  |
| Never | 185 (51) | 81 (46) | 58 (63) | 30 (41) | 16 (64) |
| Past | 128 (35) | 71 (41) | 20 (22) | 31 (42) | 6 (24) |
| Current | 53 (14) | 23 (13) | 14 (15) | 13 (18) | 3 (12) |
| *Marital status* |  |  |  |  |  |
| Single | 61 (17) | 26 (15) | 17 (19) | 12 (16) | 6 (24) |
| Married/partner | 274 (75) | 133 (76) | 70 (76) | 54 (73) | 17 (68) |
| Divorced/widowed | 30 (8) | 15 (9) | 5 (5) | 8 (11) | 2 (8) |
| *Education* |  |  |  |  |  |
| Primary | 15 (4) | 10 (6) | 3 (3) | 2 (3) | 0 (0) |
| Secondary | 60 (17) | 35 (20) | 10 (11) | 8 (11) | 7 (28) |
| Technical School | 114 (31) | 64 (37) | 20 (22) | 26 (35) | 4 (16) |
| University | 117 (48) | 66 (38) | 59 (64) | 38 (51) | 14 (56) |
| Blue-collar, yes | 95 (26) | 53 (31) | 19 (21) | 17 (23) | 6 (25) |
| bDMARDs at baseline | 175 (48) | 91 (52) | 38 (41) | 34 (46) | 12 (48) |
| BASDAI (0-10) | 4.0 (2.1) | 4.0 (2.1) | 4.0 (2.1) | 3.8 (2.2) | 4.5 (2.1) |
| BASFI (0-10) | 3.5 (2.2) | 3.3 (2.3) | 3.7 (2.3) | 3.3 (2.0) | 3.5 (2.0) |
| WPAI-presenteeism (0-100%) | 25.0 (24.6) | 22.7 (22.3) | 24.8 (24.2) | 27.8 (26.9) | 34.4 (31.8) |
| QQ-method (1-100) | 78.3 (27.2) | 80.0 (25.6) | 77.1 (29.4) | 78.3 (26.0) | 70.4 (32.9) |
| WALS (0-3) | 0.62 (0.46) | 0.59 (0.41) | 0.66 (0.53) | 0.59 (0.50) | 0.73 (0.54) |
| WLQ-25 (0-100) | 22.7 (17.7) | 23.8 (15.7) | 20.9 (21.0) | 20.4 (17.7) | 28.1 (16.1) |
| Adverse work outcome* | 22 (6) | 10 (6) | 3 (3) | 6 (8) | 3 (12) |
| Patients ≤65 years old, employed with not sick leave at baseline. Results reflect mean (SD) or n (%).  ^#^<5% missing data; ^##^<10% missing data.  *Adverse work outcome including sick leave, short-term disability and long-term disability.  axSpA, axial spondyloarthritis; BMI, body mass index; bDMARDs, biologic disease modified antirheumatic drugs; BASDAI, Bath Ankylosing Spondylitis Disease Activity Index; BASFI, Bath Ankylosing Spondylitis Functional Index; WPAI, Work Productivity and Activity Impairment; QQ-method, Quantity and Quality Method; WALS, Workplace Activity Limitations Scale; WLQ-25, Work Limitations Questionnaire. | | | | | |

| **Supplementary Table S2. Baseline characteristics of the total population and by presence of adverse work outcome during 12 months** | | | |
| --- | --- | --- | --- |
|  | Total  N=283 | No adverse work outcome  N=262 | Adverse work outcome  N=21 |
| Age, years | 43 (11) | 43 (11) | 48 (10) |
| Gender, male | 200 (71) | 187 (71) | 13 (62) |
| Caucasian race, yes | 258 (91) | 239 (91) | 19 (90) |
| Symptom duration, years^#^ | 18.2 (10.9) | 18.1 (10.9) | 19.2 (11.8) |
| Disease duration, years^#^ | 12.1 (10.2) | 12.0 (10.6) | 13.2 (10.2) |
| Pure axSpA | 80 (28) | 75 (29) | 5 (24) |
| BMI, kg/m^2 ##^ | 27.7 (5.8) | 27.7 (5.9) | 27.0 (5.6) |
| *Smoking status* |  |  |  |
| Never | 145 (51) | 137 (52) | 8 (38) |
| Past | 96 (34) | 91 (35) | 5 (24) |
| Current | 42 (25) | 34 (13) | 8 (38) |
| *Marital status* |  |  |  |
| Single | 50 (18) | 47 (18) | 3 (14) |
| Married/partner | 209 (74) | 194 (74) | 15 (71) |
| Divorced/widowed | 24 (8) | 21 (8) | 3 (14) |
| *Education* |  |  |  |
| Primary | 8 (3) | 8 (3) | 0 (0) |
| Secondary | 39 (14) | 36 (14) | 3 (14) |
| Technical School | 87 (31) | 77 (29) | 10 (48) |
| University | 149 (53) | 141 (54) | 8 (38) |
| Blue-collar, yes | 65 (23) | 60 (23) | 5 (24) |
| bDMARDs at baseline | 250 (45) | 114 (44) | 14 (67) |
| BASDAI (0-10) | 3.9 (2.0) | 3.8 (2.0) | 5.0 (2.0) |
| BASFI (0-10) | 3.3 (2.1) | 3.1 (2.1) | 4.7 (2.0) |
| WPAI-presenteeism (0-100%) | 22.4 (21.0) | 21.8 (20.8) | 30.5 (22.0) |
| QQ-method (1-100) | 90.8 (24.5) | 81.5 (24.3) | 72.4 (26.0) |
| WALS (0-3) | 0.58 (0.43) | 0.56 (0.43) | 0.86 (0.39) |
| WLQ-25 (0-100) | 22 (17) | 21 (16) | 28 (18) |
| Patients ≤65 years old, employed with not sick leave at baseline. Results reflect mean (SD) or n (%).  ^#^<5% missing data; ^##^<10% missing data.  *Adverse outcome include sick leave, short term disability and long term disability.  axSpA, axial spondyloarthritis; BMI, body mass index; bDMARDs, biologic disease modified antirheumatic drugs; BASDAI, Bath Ankylosing Spondylitis Disease Activity Index; BASFI, Bath Ankylosing Spondylitis Functional Index; WPAI, Work Productivity and Activity Impairment; QQ-method, Quantity and Quality method; WALS, Workplace Activity Limitations Scale; WLQ-25, Work Limitations Questionnaire. | | | |

| **Supplementary Table S3. Presenteeism instruments and external criteria over 12 months** | | | | | |
| --- | --- | --- | --- | --- | --- |
|  | Baseline n=366 | 3 months  n=295 | 6 months  n=295 | 9 months  n=265 | 12 months  n=237 |
| ***Presenteeism instruments*** | | | | | |
| WPAI-presenteeism (0-100%) | 25.0 (24.6) | 21.0 (22.0) | 21.3 (22.3) | 21.4 (21.0) | 21.0 (22.2) |
| QQ-method (0-100) | 78.3 (27.2) | 79.4 (27.8) | 80.9 (25.3) | 79.5 (25.7) | 78.6 (27.3) |
| WALS (0-3) | 0.62 (0.46) | 0.54 (0.42) | 0.52 (0.42) | 0.52 (0.42) | 0.52 (0.42) |
| WLQ-25 (0-100) | 22.7 (17.7) | 20.5 (17.5) | 18.2 (15.2) | 19.7 (16.9) | 19.2 (15.6) |
| BASDAI (0-10) | 4.0 (2.1) | 3.6 (2.1) | 3.6 (2.0) | 3.5 (2.1) | 3.5 (2.0) |
| BASFI (0-10) | 3.4 (2.2) | 3.1 (2.0) | 3.1 (2.1) | 3.0 (2.0) | 3.0 (2.1) |
| ***External criteria*** | | | | | |
| PAWS question | 302 (85) | 252 (87) | 243 (89) | 228 (87) | 205 (87) |
| Adverse work outcome* | 22 (6) | 14 (5) | 14 (5) | 12 (5) | 15 (6) |
| *Adverse work outcome including sick leave and short- and long-term disability.  WPAI, Work Productivity and Activity Impairment; QQ-method, Quantity and Quality method; WALS, Workplace Activity Limitations Scale; WLQ-25, Work Limitations Questionnaire; PASS, satisfactory state; BASDAI, Bath Ankylosing Spondylitis Disease Activity Index; BASFI, Bath Ankylosing Spondylitis Functional Index; PAWS, Patient Acceptable Work State. PAWS question: Considering all the different ways your disease is affecting you, if you were to stay in this state for the next few months, do you consider that your ability to perform in your current job is satisfactory? | | | | | |

| **Supplementary Table S4. ROC analysis: thresholds for excessive presenteeism according to unacceptable work state, and according four different methods of optimal cut-off determination.** | | | | | | | |
| --- | --- | --- | --- | --- | --- | --- | --- |
|  | **Optimal threshold (SE/SP)** | **N (P+N)** | **AUC**  **(95% CI)** | **75th percentile approach (95%CI)** | **Liu method (SE/ SP)** | **Youden index**  **(SE/SP)** | **Nearest to 0,1**  **(SE/SP)** |
| **WPAI presenteeism** | **40 (70/86)** | 70 (30+40) | 0.853  (0.80, 0.91) | 30 (20, 30) | **35** (70/86)  AUC 0.78 | 35 (70/86)  AUC 0.78  J=0.561 | 25 (84/71)  AUC 0.78 |
| **QQ-method*** | **3 (81/62)** | 145 (35+110) | 0.765  (0.69, 0.84) | 4.0 (4.0, 8.6) | **2.5** (81/62)  AUC 0.72 | 2.5 (81/62)  AUC 0.72  J=0.439 | **2.5** (81/62)  AUC 0.72 |
| **WALS** | **0.75 (86/75)** | 112 (37+75) | 0.870  (0.81, 0.93) | 0.75 (0.67, 0.83) | **0.74** (86/75)  AUC 0.80 | 0.74 (86/75)  AUC=0.80  J=0.607 | **0.74** (86/75)  AUC 0.80 |
| **WLQ-25** | **29 (77/80)** | 92 (33+59) | 0.853  (0.79, 0.92) | 27.2 (25.0, 29.0) | **29.0** (77/80)  AUC 0.78 | 36.8 (67/90)  AUC 0.79  J=0.579 | **29.0** (77/80)  AUC 0.78 |
| **BASDAI** | **4.7 (81/71)** | 114 (33+81) | 0.819  (0.76, 0.88) | 4.9 (4.5, 5.2) | **4.65** (81/71)  AUC 0.76 | 3.95 (91/63)  AUC=0.77  J=0.535 | **4.65** (81/71)  AUC=0.76 |
| **BASFI** | **3.5 (81/67)** | 130 (35+95) | 0.789  (0.72, 0.86) | 4.3 (3.8, 4.7) | **3.45** (81/67)  AUC 0.74 | 3.45 (81/67)  AUC=0.74  J=0.489 | **3.45** (81/67)  AUC=0.74 |
| Final optimal thresholds are bolded (in red) in the second column, as well as the corresponding number for the chosen method for the decision.  ROC, Receiver Operating Characteristic; AUC, Area Under the Curve; SE, Sensibility; SP, Specificity; WPAI, Work Productivity and Activity Impairment; QQ-method, Quantity and Quality method; WALS, Workplace Activity Limitations Scale; WLQ-25, Work Limitations Questionnaire; BASDAI, Bath Ankylosing Spondylitis Disease Activity Index; BASFI, Bath Ankylosing Spondylitis Functional Index; J, Youden index.  P+N, number of positive+negative results according to the external criterion.  AUC from Liu method, Youden index and nearest to 0.1 method correspond to the AUC of the instrument after dichotomization.  *For the analysis, the QQ-method score was inverted in order to unify the directions of all instruments. After the analysis, the result was adapted to the real score. | | | | | | | |

| **Supplementary Table S5. Performance assessment of each presenteeism instrument when classifying acceptable work state at baseline according to sex in patients with a full-time job** | | | | | | |
| --- | --- | --- | --- | --- | --- | --- |
|  | **Male (only full-time job)**  **n=231 (88%)** | | | **Female (only full-time job)**  **n=58 (56%)** | | |
|  | **Correctly classified** | **Over-estimated** | **Under-estimated** | **Correctly classified** | **Over-estimated** | **Under-estimated** |
| **WPAI**  **presenteeism ≥40** | 186 (87) | 18 (8) | 9 (4) | 44 (80) | 9 (16) | 2 (4) |
| **QQ-method <97** | 148 (69) | 60 (28) | 5 (2) | 35 (64) | 18 (33) | 2 (4) |
| **WALS ≥0.75** | 168 (79) | 39 (18) | 6 (3) | 43 (78) | 12 (22) | 0 (0) |
| **WLQ-25 ≥29** | 165 (77) | 40 (19) | 8 (4) | 46 (84) | 7 (13) | 2 (4) |
| **BASDAI ≥4.7** | 165 (77) | 44 (21) | 4 (2) | 39 (71) | 14 (25) | 2 (4) |
| **BASFI ≥3.5** | 157 (74) | 51 (24) | 5 (2) | 36 (65) | 17 (31) | 2 (4) |
| N (%)  WPAI, Work Productivity and Activity Impairment; QQ-method, Quantity and Quality method; WALS, Workplace Activity Limitations Scale; WLQ-25, Work Limitations Questionnaire; BASDAI, Bath Ankylosing Spondylitis Disease Activity Index; BASFI, Bath Ankylosing Spondylitis Functional Index. | | | | | | |

| **Supplementary Table S6. Performance assessment of each presenteeism instrument when classifying acceptable work state at 12 months** | | | | | | |  |
| --- | --- | --- | --- | --- | --- | --- | --- |
|  |  | **PAWS Acceptable work state** | **PAWS Unacceptable work state** | **Correctly classified** | **Over-estimated** | **Under-estimated** | |
| **WPAI-presenteeism ≥40** | Acceptable (<40) | 169 (96) | 7 (4) | 185 (84) | 29 (13) | 7 (3) | |
|  | Unacceptable (≥40) | 29 (64) | 16 (36) |  |  |  |  |
| **QQ-method <97** | Acceptable (≥7) | 122 (97) | 4 (3) | 141 (64) | 76 (34) | 4 (2) | |
|  | Unacceptable (<7) | 76 (80) | 19 (20) |  |  |  |  |
| **WALS ≥0.75** | Acceptable (<0.75) | 160 (96) | 6 (4) | 177 (80) | 38 (17) | 6 (3) | |
|  | Unacceptable (≥0.75) | 38 (69) | 17 (31) |  |  |  |  |
| **WLQ-25 ≥29** | Acceptable (<29) | 166 (94) | 11 (6) | 178 (81) | 32 (14) | 11 (5) | |
|  | Unacceptable (≥29) | 32 (73) | 12 (27) |  |  |  |  |
| **BASDAI ≥4.7** | Acceptable (<4.7) | 159 (96) | 7 (4) | 175 (81) | 34 (16) | 7 (3) | |
|  | Unacceptable (≥4.7) | 34 (68) | 16 (32) |  |  |  |  |
| **BASFI ≥3.5** | Acceptable (<3.5) | 147 (95) | 7 (5) | 163 (75) | 46 (21) | 7 (3) | |
|  | Unacceptable (≥3.5) | 46 (74) | 16 (26) |  |  |  |  |
| N (%)  WPAI, Work Productivity and Activity Impairment; QQ-method, Quantity and Quality method; WALS, Workplace Activity Limitations Scale; WLQ-25, Work Limitations Questionnaire; BASDAI, Bath Ankylosing Spondylitis Disease Activity Index; BASFI, Bath Ankylosing Spondylitis Functional Index. | | | | | | |  |

| **Supplementary Table S7. Performance assessment of each presenteeism instrument when classifying acceptable work state at baseline according to age** | | | | | | |
| --- | --- | --- | --- | --- | --- | --- |
|  | **≤43 years** | | | **>43 years** | | |
|  | **Correctly classified** | **Over-estimated** | **Under-estimated** | **Correctly classified** | **Over-estimated** | **Under-estimated** |
| **WPAI-**  **presenteeism ≥40** | 143 (87) | 17 (10) | 4 (2) | 140 (81) | 23 (13) | 9 (5) |
| **QQ-method <97** | 108 (66) | 53 (32) | 3 (2) | 110 (64) | 57 (33) | 5 (3) |
| **WALS ≥0.75** | 129 (79) | 31 (19) | 4 (2) | 126 (73) | 44 (26) | 2 (1) |
| **WLQ-25 ≥29** | 131 (80) | 28 (17) | 4 (2) | 135 (78) | 31 (18) | 6 (3) |
| **BASDAI ≥4.7** | 115 (70) | 45 (27) | 4 (2) | 130 (76) | 36 (21) | 6 (3) |
| **BASFI ≥3.5** | 124 (76) | 35 (21) | 5 (3) | 108 (63) | 60 (35) | 3 (2) |
| N (%)  WPAI, Work Productivity and Activity Impairment; QQ-method, Quantity and Quality method; WALS, Workplace Activity Limitations Scale; WLQ-25, Work Limitations Questionnaire; BASDAI, Bath Ankylosing Spondylitis Disease Activity Index; BASFI, Bath Ankylosing Spondylitis Functional Index. | | | | | | |


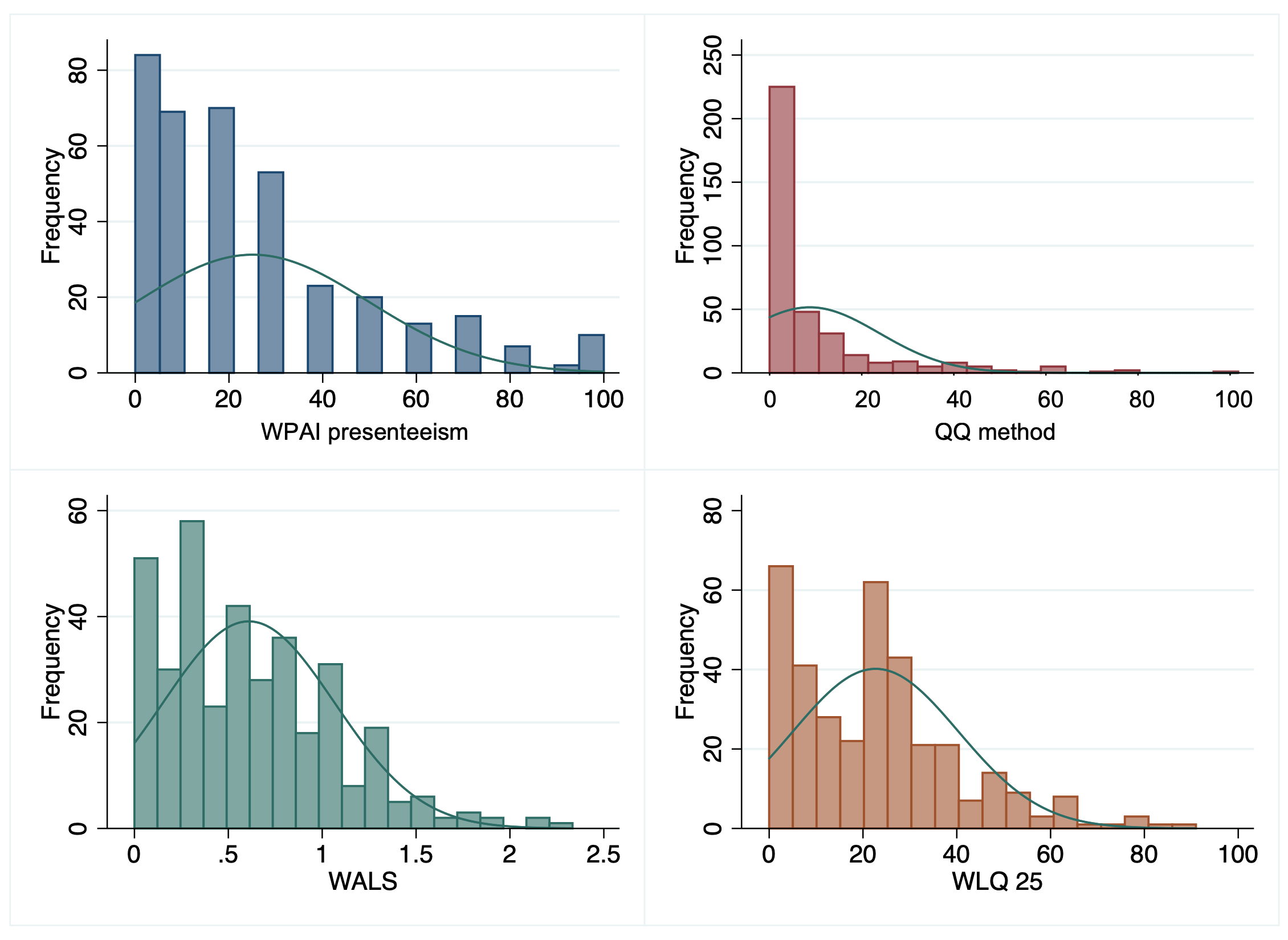


**Supplementary Figure S1. Distribution of the four presenteeism instruments.**

| **Supplementary Table S8. Performance assessment of each presenteeism instrument when classifying acceptable work state at baseline according to sex** | | | | | | |
| --- | --- | --- | --- | --- | --- | --- |
|  | **Male** | | | **Female** | | |
|  | **Correctly classified** | **Over-estimated** | **Under-estimated** | **Correctly classified** | **Over-estimated** | **Under-estimated** |
| **WPAI-presenteeism ≥40** | 211 (87) | 21 (9) | 11 (5) | 72 (77) | 19 (20) | 2 (2) |
| **QQ-method <97** | 166 (68) | 71 (29) | 6 (2) | 52 (56) | 39 (42) | 2 (2) |
| **WALS ≥0.75** | 189 (78) | 48 (20) | 6 (2) | 66 (71) | 27 (29) | 0 (0) |
| **WLQ-25 ≥29** | 188 (77) | 47 (19) | 8 (3) | 78 (85) | 12 (13) | 2 (2) |
| **BASDAI ≥4.7** | 185 (76) | 52 (21) | 6 (2) | 60 (65) | 29 (31) | 4 (4) |
| **BASFI ≥3.5** | 176 (72) | 62 (26) | 5 (2) | 56 (61) | 33 (36) | 3 (3) |
| N (%)  WPAI, Work Productivity and Activity Impairment; QQ-method, Quantity and Quality method; WALS, Workplace Activity Limitations Scale; WLQ-25, Work Limitations Questionnaire; BASDAI, Bath Ankylosing Spondylitis Disease Activity Index; BASFI, Bath Ankylosing Spondylitis Functional Index. | | | | | | |

| **Supplementary Table S9. Performance assessment of each presenteeism instrument when classifying acceptable work state at baseline according to job type** | | | | | | |
| --- | --- | --- | --- | --- | --- | --- |
|  | **Blue-collar** | | | **White-collar** | | |
|  | **Correctly classified** | **Over-estimated** | **Under-estimated** | **Correctly classified** | **Over-estimated** | **Under-estimated** |
| **WPAI-**  **presenteeism ≥40** | 65 (76) | 14 (16) | 6 (7) | 212 (87) | 26 (11) | 6 (2) |
| **QQ-method <97** | 46 (54) | 36 (42) | 3 (4) | 165 (68) | 74 (30) | 5 (2) |
| **WALS ≥0.75** | 66 (78) | 17 (20) | 2 (2) | 185 (76) | 56 (23) | 3 (1) |
| **WLQ-25 ≥29** | 66 (78) | 14 (16) | 5 (6) | 196 (81) | 43 (18) | 4 (2) |
| **BASDAI ≥4.7** | 59 (69) | 23 (27) | 3 (4) | 181 (74) | 56 (23) | 7 (3) |
| **BASFI ≥3.5** | 58 (68) | 25 (29) | 2 (2) | 169 (70) | 68 (28) | 6 (2) |
| N (%)  WPAI, Work Productivity and Activity Impairment; QQ-method, Quantity and Quality method; WALS, Workplace Activity Limitations Scale; WLQ-25, Work Limitations Questionnaire; BASDAI, Bath Ankylosing Spondylitis Disease Activity Index; BASFI, Bath Ankylosing Spondylitis Functional Index. | | | | | | |

| **Supplementary Table S10. Performance assessment of each presenteeism instrument when classifying acceptable work state at baseline according to type of disease** | | | | | | |
| --- | --- | --- | --- | --- | --- | --- |
|  | **Pure axSpA** | | | **axSpA with peripheral involvement** | | |
|  | **Correctly classified** | **Over-estimated** | **Under-estimated** | **Correctly classified** | **Over-estimated** | **Under-estimated** |
| **WPAI-**  **presenteeism ≥40** | 79 (84) | 11 (12) | 4 (4) | 204 (84) | 29 (12) | 9 (4) |
| **QQ-method <97** | 63 (67) | 29 (31) | 2 (2) | 155 (64) | 81 (33) | 6 (2) |
| **WALS ≥0.75** | 75 (80) | 15 (16) | 4 (4) | 180 (74) | 60 (25) | 2 (1) |
| **WLQ-25 ≥29** | 76 (81) | 14 (15) | 4 (4) | 190 (79) | 45 (19) | 6 (2) |
| **BASDAI ≥4.7** | 75 (80) | 17 (18) | 2 (2) | 170 (70) | 64 (26) | 8 (3) |
| **BASFI ≥3.5** | 69 (73) | 23 (24) | 2 (2) | 163 (68) | 72 (30) | 6 (2) |
| N (%)  WPAI, Work Productivity and Activity Impairment; QQ-method, Quantity and Quality method; WALS, Workplace Activity Limitations Scale; WLQ-25, Work Limitations Questionnaire; BASDAI, Bath Ankylosing Spondylitis Disease Activity Index; BASFI, Bath Ankylosing Spondylitis Functional Index. | | | | | | |

| **Supplementary Table S11. Performance assessment of each presenteeism instrument when classifying acceptable work state at baseline according to education** | | | | | | |
| --- | --- | --- | --- | --- | --- | --- |
|  | **No superior education** | | | **Superior education** | | |
|  | **Correctly classified** | **Over-estimated** | **Under-estimated** | **Correctly classified** | **Over-estimated** | **Under-estimated** |
| **WPAI-**  **presenteeism ≥40** | 49 (74) | 12 (18) | 5 (8) | 234 (87) | 28 (10) | 8 (3) |
| **QQ-method <97** | 35 (53) | 27 (41) | 4 (6) | 183 (68) | 83 (31) | 4 (1) |
| **WALS ≥0.75** | 51 (77) | 14 (21) | 1 (2) | 204 (76) | 61 (23) | 5 (2) |
| **WLQ-25 ≥29** | 48 (73) | 13 (20) | 5 (8) | 218 (81) | 46 (17) | 5 (2) |
| **BASDAI ≥4.7** | 45 (68) | 18 (27) | 3 (5) | 200 (74) | 63 (23) | 7 (3) |
| **BASFI ≥3.5** | 39 (60) | 25 (38) | 1 (2) | 193 (71) | 70 (26) | 7 (3) |
| N (%)  WPAI, Work Productivity and Activity Impairment; QQ-method, Quantity and Quality method; WALS, Workplace Activity Limitations Scale; WLQ-25, Work Limitations Questionnaire; BASDAI, Bath Ankylosing Spondylitis Disease Activity Index; BASFI, Bath Ankylosing Spondylitis Functional Index. | | | | | | |

| **Supplementary Table S12. Performance assessment of each presenteeism instrument when classifying acceptable work state at baseline according to BMI** | | | | | | |
| --- | --- | --- | --- | --- | --- | --- |
|  | **BMI ≤28** | | | **BMI >28** | | |
|  | **Correctly classified** | **Over-estimated** | **Under-estimated** | **Correctly classified** | **Over-estimated** | **Under-estimated** |
| **WPAI-**  **presenteeism ≥40** | 160 (85) | 23 (12) | 6 (3) | 123 (84) | 17 (12) | 7 (5) |
| **QQ-method <97** | 120 (63) | 64 (34) | 5 (3) | 98 (67) | 46 (31) | 3 (2) |
| **WALS ≥0.75** | 149 (79) | 36 (19) | 4 (2) | 106 (72) | 39 (27) | 2 (1) |
| **WLQ-25 ≥29** | 155 (82) | 29 (15) | 5 (3) | 111 (76) | 30 (21) | 5 (3) |
| **BASDAI ≥4.7** | 134 (71) | 50 (26) | 5 (3) | 111 (76) | 31 (21) | 5 (3) |
| **BASFI ≥3.5** | 137 (72) | 47 (25) | 5 (3) | 95 (65) | 48 (33) | 3 (2) |
| N (%)  BMI, body mass index; WPAI, Work Productivity and Activity Impairment; QQ-method, Quantity and Quality method; WALS, Workplace Activity Limitations Scale; WLQ-25, Work Limitations Questionnaire; BASDAI, Bath Ankylosing Spondylitis Disease Activity Index; BASFI, Bath Ankylosing Spondylitis Functional Index. | | | | | | |

| **Supplementary Table S13. Performance assessment of each presenteeism instrument when classifying acceptable work state at baseline according to symptom duration (<18 years or ≥18 years)** | | | | | | |
| --- | --- | --- | --- | --- | --- | --- |
|  | **Symptom duration <18 years** | | | **Symptom duration ≥18 years** | | |
|  | **Correctly classified** | **Over-estimated** | **Under-estimated** | **Correctly classified** | **Over-estimated** | **Under-estimated** |
| **WPAI-**  **presenteeism ≥40** | 137 (84) | 19 (12) | 7 (4) | 146 (84) | 21 (12) | 6 (3) |
| **QQ-method <97** | 101 (62) | 59 (36) | 3 (2) | 117 (68) | 51 (29) | 5 (3) |
| **WALS ≥0.75** | 119 (73) | 38 (23) | 6 (4) | 136 (79) | 37 (21) | 0 (0) |
| **WLQ-25 ≥29** | 122 (75) | 35 (21) | 6 (4) | 144 (83) | 24 (14) | 4 (2) |
| **BASDAI ≥4.7** | 117 (72) | 44 (27) | 2 (1) | 128 (74) | 37 (21) | 8 (5) |
| **BASFI ≥3.5** | 119 (73) | 39 (24) | 5 (3) | 113 (66) | 56 (33) | 3 (2) |
| N (%)  WPAI, Work Productivity and Activity Impairment; QQ-method, Quantity and Quality method; WALS, Workplace Activity Limitations Scale; WLQ-25, Work Limitations Questionnaire; BASDAI, Bath Ankylosing Spondylitis Disease Activity Index; BASFI, Bath Ankylosing Spondylitis Functional Index. | | | | | | |

| **Supplementary Table S14. Baseline characteristics of completers and patients who dropped-out between baseline and 12 months.** | | | |
| --- | --- | --- | --- |
|  | Completers  N=246 | Dropped-out patients  N=120 | p-value |
| Age, years | 44 (10) | 42 (11) | 0.137 |
| Gender, male | 177 (72) | 86 (72) | 0.955 |
| Caucasian | 227 (92) | 110 (92) | 0.839 |
| Symptom duration, years^#^ | 18.9 (0.7) | 16.9 (1.0) | 0.108 |
| Disease duration, years^#^ | 12.2 (0.6) | 10.3 (0.9) | 0.081 |
| Pure axSpA | 72 (29) | 28 (23) | 0.142 |
| BMI, kg/m^2 ##^ | 27.6 (0.4) | 29.0 (0.8) | 0.112 |
| *Smoking status* |  |  | 0.504 |
| Never | 120 (49) | 65 (54) |  |
| Past | 91 (37) | 37 (31) |  |
| Current | 35 (14) | 18 (15) |  |
| *Marital status* |  |  | 0.705 |
| Single | 39 (16) | 22 (18) |  |
| Married/partner | 185 (75) | 89 (75) |  |
| Divorced/widowed | 22 (9) | 8 (7) |  |
| *Education* |  |  | 0.082 |
| Primary | 6 (2) | 9 (8) |  |
| Secondary | 37 (15) | 23 (19) |  |
| Technical School | 78 (32) | 36 (30) |  |
| University | 125 (51) | 52 (43) |  |
| Blue-collar, yes | 60 (25) | 35 (30) | 0.303 |
| bDMARDs at baseline | 112 (46) | 63 (52) | 0.127 |
| BASDAI (0-10) | 3.95 (2.05) | 4.08 (2.32) | 0.594 |
| BASFI (0-10) | 3.40 (2.21) | 3.47 (2.25) | 0.782 |
| WPAI-presenteeism (0-100%) | 24.55 (24.37) | 26.08 (25.15) | 0.577 |
| QQ-method (1-100) | 78.03 (27.14) | 78.77 (27.46) | 0.966 |
| WALS (0-3) | 0.60 (0.46) | 0.65 (0.47) | 0.300 |
| WLQ-25 (0-100) | 21.82 (17.33) | 24.43 (18.29) | 0.192 |
| Adverse work outcome* | 16 (6) | 6 (5) | 0.378 |
| Patients ≤65 years old, employed at baseline. Results reflect mean (SD) or n (%).  ^#^<5% missing data; ^##^<10% missing data.  *Adverse work outcome including sick leave, short-term disability and long-term disability.  axSpA, axial spondyloarthritis; BMI, body mass index; bDMARDs, biologic disease modified antirheumatic drugs; BASDAI, Bath Ankylosing Spondylitis Disease Activity Index; BASFI, Bath Ankylosing Spondylitis Functional Index; WPAI, Work Productivity and Activity Impairment; QQ-method, Quantity and Quality Method; WALS, Workplace Activity Limitations Scale; WLQ-25, Work Limitations Questionnaire. | | | |
